# Extreme Nipple-Sparing Mastectomy: Feasibility of Nipple Preservation and Immediate Reconstruction in Breasts Weighing Over 600 Grams in a Cohort of 43 Patients

**DOI:** 10.1155/tbj/6974079

**Published:** 2025-02-22

**Authors:** Vaishali Purohit, Jasmine Dwyer, Andrea Moreira, Jenna Li, Emil Fernando, Janette Gomez, Jennifer Saldanha, Thomas Julian, Suzanne Coopey

**Affiliations:** ^1^Division Breast Surgical Oncology, Department of Surgery, Allegheny Health Network, Pittsburgh, Pennsylvania, USA; ^2^Division of Plastic Surgery, Department of Surgery, University of Pittsburgh Medical Center, Pittsburgh, Pennsylvania, USA; ^3^Cancer Institute, Allegheny Health Network Research Institute, Pittsburgh, Pennsylvania, USA; ^4^Department of Surgery, Drexel University College of Medicine, Philadelphia, Pennsylvania, USA

**Keywords:** immediate reconstruction, large-volume breast, nipple-sparing mastectomy, postoperative complications

## Abstract

**Background:** Limited data exist on complication rates in nipple-sparing mastectomy (NSM) in patients with large-volume breasts. Our aim was to evaluate the early complication rates of NSM with immediate reconstruction in a consecutive cohort of patients with large-volume breasts.

**Methods:** After IRB approval, patients treated with prophylactic or therapeutic NSM and immediate reconstruction from January 2020 to June 2022 at our health network were identified. Patients with breast weights > 600 g (the extreme NSM group) were compared to patients with breast weights of 600 g or less (the average-volume NSM group).

**Results:** A total of 184 patients underwent NSM with immediate reconstruction. Forty-three of 184 (23.37%) NSM patients had breast weights > 600 g. Of these, 30 patients had bilateral NSM, for a total of 73 breasts with volumes over 600 g, ranging from 603 to 1658 g. There were significantly more total complications in the extreme NSM compared to average-volume NSM groups (41.86% vs. 21.99%, *p*=0.009852). When broken down into major and minor complications, the extreme NSM group had significantly more major complications than the average-volume NSM group (27.91% vs. 12.86%, *p*=0.01072), but no difference in minor complications (13.95% vs. 9.29%, *p*=0.2205). Overall, one (2.33%) patient in the extreme NSM group had a reconstruction failure, compared to three (2.14%) in the average-volume NSM group. Only two of 43 (4.65%) patients in the extreme NSM group lost their nipples due to total or partial nipple necrosis.

**Conclusions:** NSM with immediate reconstruction was successful in the majority of patients with large-volume breasts. The rate of nipple loss was acceptably low. Women with breast volumes larger than 600 g who are motivated to save their nipples at the time of mastectomy could be offered NSM.

## 1. Introduction

Nipple-sparing mastectomy (NSM) has been shown to improve quality of life, improve patient satisfaction with aesthetic outcomes, and decrease body image disturbance [1]. The oncologic safety of NSM is comparable to simple and skin-sparing mastectomy based on a multitude of studies, and the risk of nipple recurrence after NSM is very low [1, 2]. With careful tissue handling and preservation of perfusion to mastectomy skin flaps, an increasing number of oncologic patients are now being offered NSM [2, 3]. Patients with larger breasts inevitably have longer skin flaps and a farther distance for the blood supply to travel to reach the nipple. Postoperative partial or total necrosis of the nipple–areola complex (NAC) is a unique complication to NSM; it has been historically reported to occur in up to 60% [4], but only in up to 10% of cases in more recent literature [5, 6]. While partial, or superficial, nipple necrosis is quite common and typically heals without long-term problems, total, or full-thickness, nipple necrosis often leads to nipple loss and can, in rare circumstances, be a nidus for infection and even reconstruction loss.

Selecting patients to optimize the outcomes and reduce the risk of complications, such as skin and NAC necrosis, often exempts patients with large-volume breasts from consideration for NSM [3]. Initial guidelines excluded patients with previous radiation, breast ptosis, high body mass index (BMI), and macromastia with breast size over 500 g [3]. Despite data supporting the oncologic safety of NSM in appropriate candidates, concerns still remain about the feasibility of NSM with immediate reconstruction in patients with large-volume breasts due to longer length of skin flaps and possible blood supply issues theoretically leading to ischemic sequelae with reconstruction [3, 7, 8]. More recently, stringent criteria that limited NSM to women with favorable characteristics, such as small breast size, are being challenged as surgical teams gain expertise with NSM [9]; however, limited data exist on complication rates in NSM in large-volume breasts, or what we describe here as extreme NSM.

Our aim was to evaluate the early complication rates of NSM with immediate reconstruction in large-volume breasts over 600 g compared to those with breast weights of 600 g or less. We hypothesize that women with larger volume breasts would have a small increased the risk of early complications but that the risk of nipple loss due to total necrosis would be low.

## 2. Methods

After institutional IRB approval, consecutive patients who were treated with prophylactic or therapeutic NSM plus immediate reconstruction from January 2020 to June 2022 at Allegheny Health Network were identified. NSM patients were separated into two cohorts for comparison: patients with breast weights > 600 g (the extreme NSM group) and patients with breast weights of 600 g or less (the average-volume NSM group). Breast weight was available in the operative report and/or pathology report. The 600-g threshold was chosen because it represents a breast size larger than what most surgeons would typically consider suitable for NSM, reflecting a group at the outer limits of the current practice criteria.

Patients underwent NSM by seven different breast surgeons and immediate reconstruction by six different plastic surgeons. Surgeries were performed in both academic teaching hospitals and community hospitals, all within Allegheny Health Network. Whether or not a patient was a candidate for NSM was jointly decided by the breast surgeon and plastic surgeon. Reconstruction types included tissue expander, direct to implant, and autologous reconstruction with deep inferior epigastric perforator (DIEP) flaps. Some patients were offered skin-only mastopexy at the time of reconstruction as previously described by Aliotta [10], upon discretion of the plastic surgeon. NSM was performed to remove the breast tissue through seven different incisions: transverse, inferior vertical (at 6:00 position), circumareolar, lateral radial, inframammary, inferolateral, and Wise pattern.

Patient demographics, intraoperative variables, and postoperative complications were collected from the electronic medical record and analyzed in September–October 2022. This time period allowed for at least 3 months of follow-up to assess for early postoperative complications. All patient postoperative notes by breast surgeon and plastic surgeon were reviewed for documentation of complications. Any additional hospital admissions, operations, radiology procedures, and/or microbiology cultures were captured. Major and minor complications were evaluated for each patient, including the development of seroma, hematoma, wound dehiscence, wound infection, skin flap necrosis, total nipple necrosis, partial DIEP flap necrosis, total DIEP flap necrosis, capsular contracture, implant displacement, implant extrusion, implant rupture, fat necrosis, and cellulitis. Major complications were defined as those requiring a return to the operating room for intervention, while minor complications were defined as those managed without additional surgery. If a patient had both a minor and major complication, they were included in the major complication group only. Seromas and hematomas were diagnosed if they were documented in a postoperative note on clinical exam, if there was imaging evidence on ultrasound or CT scan, and/or if the patient underwent an aspiration or surgical evacuation procedure. Partial necrosis implied that only a portion of the DIEP flap was lost due to a perfusion problem, while total necrosis indicated a complete flap loss. If there was no documentation of a particular complication in the electronic medical record, it was assumed to be absent.

Since this study was a retrospective review with minimal risk to patients, our IRB deemed it exempt from requiring patient consent. However, all patients did consent to the use of photography for scientific purposes, provided that their identity was not revealed by the pictures or by the descriptive text accompanying them.

### 2.1. Statistical Analysis

To compare the extreme NSM group and the average-volume NSM group, two-sided *t*-tests were used for comparison of continuous variables, and chi-squared tests or Fisher's exact tests were used to compare categorical variables. Significance was determined at an alpha level of 0.05.

## 3. Results

### 3.1. Patient Characteristics

From January 2020 to June 2022, a total of 184 patients underwent NSM with immediate reconstruction at Allegheny Health Network. Forty-three of 184 (23.4%) NSM patients had breast weights over 600 g and were included in the extreme NSM group. Of these, 30 patients had bilateral mastectomies, for a total of 73 breasts with breast volume over 600 g, ranging from 603 g to 1658 g. One hundred and forty-one patients were in the average-volume NSM group. Of these, 104 patients had bilateral NSM, for a total of 245 breasts weighing ≤ 600 g.

There were no significant differences found in age, race, menopausal status, cancer stage, radiation use, neoadjuvant chemotherapy, diabetic status, tobacco use, or reconstruction type between patients who had extreme and average-volume NSM (Table 1). The patients in the extreme NSM group had significantly higher mean BMI, larger mean initial implant size, and higher proportion of patients with a history of hypertension compared to the average-volume NSM group. The mean BMI in average-volume NSM was 24.9 (range 17.5–43.88), while the mean BMI for extreme NSM was 32.02 (range 23–46) (*p* < 0.05).

### 3.2. Surgery Details

**Figure 1:**
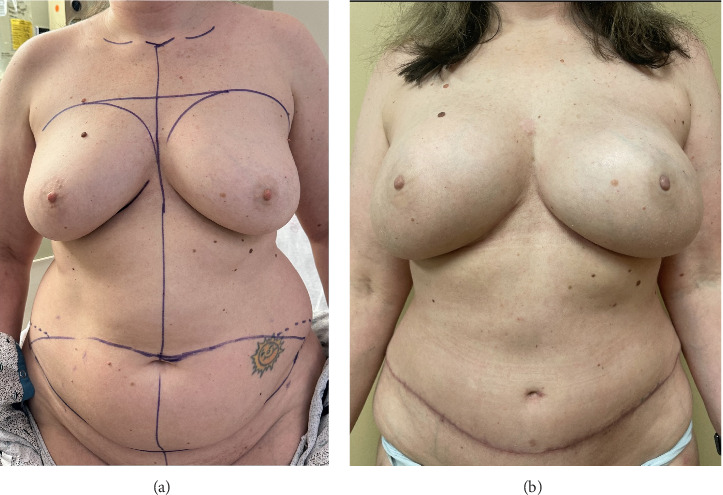
(a) 36-year-old patient, BRCA 2, who underwent bilateral prophylactic NSM and DIEP reconstruction, mastectomy specimen 648 g. (b) Postoperative outcome after revision.

**Figure 2:**
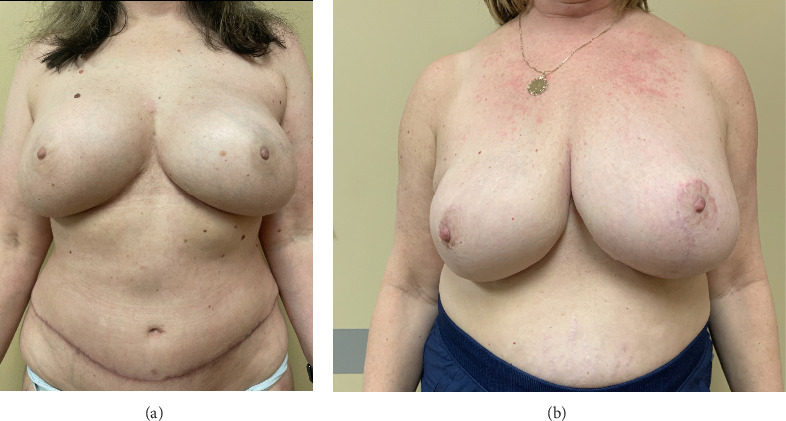
(a) Sixty-one year old patient with right breast cancer, s/p right nipple sparing mastectomy with skin-only mastopexy and direct-to-implant reconstruction. Right mastectomy specimen 1480 g and left reduction specimen 990 g. (b) Postoperative outcome after a second stage with revision and fat grafting.

In both the average-volume NSM group and extreme NSM group, the majority of patients underwent direct-to-implant reconstruction (41.84% and 53.49%, respectively) (Table 2). 41.84% of average-volume NSM patients underwent expander placement as the first step of reconstruction, compared to 53.49% of extreme NSM patients. 17.02% of average-volume NSM patients underwent DIEP flap reconstruction, compared to 18.6% of extreme NSM patients (Figure 1). For patients who underwent direct-to-implant reconstruction, the mean implant size for extreme NSM was 608.93 mL, and the mean implant size for average-volume NSM was 396.61 mL, which was a statistically significant difference (*p* < 0.05) (Figure 2).

### 3.3. Complications

At a mean follow-up of 142 days and 151 days for average-volume NSM and extreme NSM, respectively (range 6–753 days), there was a significantly increased total complication rate in the extreme NSM group compared to the average-volume NSM group (41.86% vs. 21.43%, *p*=0.0077). When broken down into major and minor complications, the extreme NSM group had significantly more major complications than the average-volume NSM group (27.91% vs. 12.14%, *p*=0.01072), but no difference in minor complications (13.95% vs. 9.29%, *p*=0.2205) (Table 3).

Rates of seroma, hematoma, skin flap necrosis, nipple necrosis, wound dehiscence, wound infection, partial flap necrosis, and implant extrusion were numerically slightly higher in the extreme NSM group compared to average-volume NSM group, as noted in Table 4. The number of events in each of these groups was too few for statistical analysis, and patients often developed multiple simultaneous complications (for instance, skin flap necrosis leading to wound dehiscence and/or infection). There was a higher incidence of cellulitis in the average-volume NSM group compared to the extreme NSM group.

Only two of 43 (4.65%) patients in the extreme NSM group lost their nipples due to total or partial nipple necrosis (Table 4). One of the two patients had NSM with direct-to-implant reconstruction and required take back for complete nipple excision in the operating room. The second of the two patients had NSM with DIEP flap reconstruction; she had nipple necrosis requiring Silvadene application and local debridement in the clinic only. Overall, one (2.32%) patient in the extreme NSM group had a reconstruction failure, compared to three (2.13%) patients in the average-volume NSM group.

## 4. Discussion

### 4.1. Key Findings

Despite having a significantly higher risk of early postoperative complications, NSM with immediate reconstruction was successful for most patients with large-volume breasts over 600 g. Of the 43 patients having 73 NSM plus immediate reconstruction, 42 of the 43 patients (97.67%) had successful immediate reconstruction, with only one reconstruction failure. This is similar to the rate of patients with successful reconstruction in the average-volume NSM group, which was 138 out of 141 patients (97.8%). Only two of 43 (4.65%) patients in the extreme NSM group lost their nipples due to total nipple necrosis.

### 4.2. Strengths and Limitations

Although our sample size is small, this would be one of the largest series in the literature with 73 NSM in breasts weighing more than 600 g. Because this is an observational cohort study, there is a definite selection bias. Patients with large breasts were offered NSM when the plastic surgeon and breast surgeon thought it was feasible. This decision was likely based on many factors including plastic surgeon and breast surgeon experience with this technique, plastic surgeon and breast surgeon compatibility and familiarity with each other, and patient risk factors such as prior radiation, smoking, BMI, and diabetes. Because this is a retrospective study, complications were gathered from the electronic medical record. If documentation was lacking or incomplete, this may have led to an underestimation of minor complications.

Our study is limited in its ability to evaluate the effects of postmastectomy radiation on complications due to the relatively short follow-up period. Longer-term follow-up is necessary to fully assess the impact of radiation on late complications, including capsular contracture, implant extrusion, and reconstruction failure. Additionally, our study is limited by the absence of patient-reported outcomes, which are critical for understanding the quality of life and cosmetic satisfaction.

### 4.3. Comparison to Similar Research

Several factors have been shown in the literature to increase early breast reconstruction complications, including body mass index [11, 12]. Davies et al. reported a higher risk of complications after skin-sparing mastectomy in women with a BMI greater than 25 kg/m^2^ [13]. In the study reported here, we did find that patients in the extreme NSM group also had a statistically higher rate of comorbidities, such as higher BMI and hypertension, which likely contributed to increased complications in this group. The overall complication rate in our study for average-volume NSM was 21.43%, compared to 41.86% in extreme NSM. This is comparable to the overall complication rate of 35% in 68 patients undergoing NSM in the literature, in a study that did not include a specific cohort of large-volume breasts [1].

Several comorbidities have been discussed in the literature as relative contraindications to NSM, such as high BMI and macromastia. A study by Metere et al., which included 894 NSM patients, found nipple necrosis in 6.4% of patients [14]. Of note, the majority of patients in their study had BMI < 30, while the average BMI of the patients in our study with extreme NSM was over 30 [14]. In addition, macromastia with breast size over 500 g has also been reported as a relative contraindication to offering NSM [3]. However, our data demonstrate that the majority of patients with breast volume greater than 600 g, 95% (41/43 patients), did not have NAC necrosis. This is similar to the NAC preservation rate in a study by de Alcantara Filho et al., who demonstrated that the NAC was entirely preserved in 96.7% of patients undergoing NSM at Memorial Sloan Kettering Cancer Center [2], even though the exclusion criteria in this study excluded patients with Grade 4 ptosis, oversized breasts, diabetes, heavy smoking history, and obesity.

Additionally, Mitchell et al. reported a NAC complication rate of 4.5%, defined as necrosis or other ischemia requiring surgery, in a dataset of the American Society of Breast Surgeons Nipple Sparing Mastectomy Registry representing 1935 NSMs with different breast sizes and using different stages of reconstruction, which is similar to the rate of NAC necrosis of 4.88% reported here in the extreme NSM group with immediate reconstruction [15].

Few studies have included patients with large breast volume, which makes this study unique. Jensen et al. demonstrated that the majority of the 8/127 (6.3%) patients with nipple necrosis and subsequent nipple removal during the early postoperative period in his study had ptotic breasts [3, 16]. A study reported by Wang et al. found that a group of patients with larger breasts over 352 g had an 8.1% increase in the risk of superficial nipple necrosis compared to a group with smaller breasts under 352 g. Patients in the larger group were also noted to have a higher BMI, similar to our study [17].

In a study of 124 NSM, Chirappapha et al. reported patients with a breast resection volume larger than 750 cm^3^ had 23% NAC necrosis, compared to 6% NAC necrosis in patients with less than 750 cm^3^ of removed breast volume [18]. Our data demonstrate nipple necrosis rates much lower than this. Chirappapha also observed a trend of higher risk of nipple necrosis in ptotic breasts, with a larger volume of breast volume removed, and a larger volume of prosthesis inserted for the reconstruction, but the findings were not statistically significant [18].

Additionally, a study by Frey et al., which stratified patients by mastectomy weights of greater than 800 g, 400–799 g, and less than 400 g, found complete NAC necrosis rates to reach 12.1%, 2.7%, and 1%, respectively [7]. Compared to this, the data in our study showed a nipple necrosis rate of 4.65%, in breast sizes ranging from 603 g to 1658 g.

Increased rates of skin flap necrosis in large-volume mastectomies have also been reported in the literature, with one study reporting that cases with mastectomy specimen mass more than 600 g had 10.4 times the risk of skin necrosis requiring debridement [17]. Wang et al. demonstrated that the larger breast group had a 4.3% higher risk of minor skin necrosis, but no difference in the risk of major skin necrosis [17]. In our study, the extreme NSM group had a rate of 6.98% of skin flap necrosis, compared to 2.12% in the average-volume NSM group. Of note, both groups had very few patients and therefore could not be effectively compared statistically.

Previously, breast size measured by cup size and BMI were shown to be associated with skin flap necrosis, and more recently, Masten showed that larger mastectomy specimen size is associated with moderate to severe skin flap necrosis, with an overall rate of skin flap necrosis after mastectomy with reconstruction of 14% [19]. Our data showed a lower rate of skin flap necrosis even for the extreme NSM group.

Furthermore, wound infection is a significant concern after NSM listed in the literature. An overall infection rate of 4.4% was noted by Mitchell et al. in NSM of all volumes [15]. The wound infection rate for both average-volume and extreme NSM was below 5% in our study, which is comparable to the American Society of Breast Surgeons' Registry study.

Other techniques have been described to expand NSM eligibility to patients with ptosis and/or larger breasts. One such procedure reported by Spear et al. is a staged NSM, whereby a mastopexy or reduction is completed weeks prior to NSM, in an attempt to improve vascularity [20]. Utilizing this technique, Spear et al. reported a partial NAC necrosis rate of 13.9% and a mastectomy flap necrosis rate of 16.3% [20]. Another described procedure is the delay technique, which involves the dissection of the tissue beneath the NAC to separate the NAC from underlying breast parenchyma weeks prior to the NSM procedure [21, 22]. This was first reported by Palmieri et al. and further described by Halsey et al. and Lee et al. Lee et al. reported that the delayed NSM group had higher total and major complication rates than the NSM-alone group, although not statistically significant [21]. NAC complications in the delayed NSM group remained low (18.1% patients, 11.9% nipples), and similar to the NSM-alone group (15.5% patients, 9.3% nipples) [21]. There was no NAC loss in the delayed NSM group in their study, compared to the 2.9% NAC loss in the NSM-alone group [21]. In their study, however, the delayed NSM group had higher ptosis grades, as well as higher mastectomy weight specimens than the NSM-alone group, with the delayed group weight being 503.9 ± 193.8 [21]. Our study had higher breast volume in a single-stage procedure and had similarly low complication rates. Although demonstrated to be safe, limitations of the delayed NSM techniques after possible mastopexy include adding additional surgical procedures to the patient's clinical course, the possibility of delaying final treatment, and the potential of creating scars that may jeopardize NAC viability with NSM [20–22]. For this reason, we do believe it is important to have the ability to provide a single-stage NSM procedure, even to patients with large breast volume, as reported in this study.

### 4.4. Explanation of Findings

Early studies with NSM demonstrated high rates of ischemic complications involving the nipple and mastectomy skin flaps, despite careful patient selection [3–6]. Over time, as the technique became more refined and surgeons became more experienced, the rate of ischemic complications has improved, with more recent studies showing rates of nipple necrosis under 10% [11, 23]. Specifically, the dissection of the nipple involves mastery of a technique, which carefully preserves the nipple blood supply without compromising oncologic safety. For that, the surgeons in our group performed a sharp dissection of the nipple with minimal tissue trauma.

Additionally, the inferolateral incision, or a laterally based inframammary incision, is preferred by the surgeons at our institution in recent years, as it allows for preservation of the medial branches of the fifth intercostal perforator, which has been shown to contribute to the perfusion of the mastectomy skin flap and nipple [24, 25].

### 4.5. Implications

Nipple preservation in patients with large-volume breasts requires close collaboration between the breast oncologic surgeon and plastic surgeon. Placing an inferolateral incision and carefully dissecting the anterior skin flap to preserve blood supply are critical to the success of this procedure. There is no harm in attempting nipple preservation; if the nipple looks ischemic after the completion of mastectomy, the mastectomy can simply be converted to skin-sparing if needed.

## 5. Conclusion

When given the option, most patients having mastectomy would prefer an attempt at nipple preservation. In our experience, most patients with larger volume breasts can successfully undergo NSM with immediate reconstruction. Despite an increased risk of complications, rates of nipple loss are acceptably low and most complications resolve without leading to reconstruction failure.

## Figures and Tables

**Table 1:** Demographics and preoperative characteristics of patients undergoing average-volume versus extreme NSM with immediate reconstruction.

Variable	Average-volume NSM (< 600 g)	Extreme NSM (> 600 g)	*p* value
Total number of patients	141	43	
Total number of breasts	245	73	
Mean age ± SD, yr	47.67 ± 8.96	48.33 ± 11.24	0.7255
Mean BMI ± SD, kg/m^2^	24.9 ± 4.26	32.02 ± 5.37	**0**
Mean breast weight ± SD, grams	321.2 ± 129.29	831.73 ± 296.79	**0**
Mean initial implant size ± SD, milliliters	396.61 ± 120.95	608.93 ± 111.42	**0**
Diabetes			
No (%)	137 (97.16)	40 (93.02)	0.3569
Yes (%)	4 (2.84)	3 (6.98)	
Hypertension			
No (%)	119 (84.4)	29 (67.44)	**0.01415**
Yes (%)	22 (15.6)	14 (32.56)	
ASA class			
Class 1 (%)	14 (9.93)	0 (0)	0.07234
Class 2 (%)	99 (70.21)	35 (81.4)	
Class 3 (%)	28 (19.86)	8 (18.6)	
Prophylactic			
No (%)	110 (78.01)	38 (88.37)	0.1339
Yes (%)	31 (21.99)	5 (11.63)	
Laterality			
Unilateral (%)	37 (26.24)	13 (30.23)	0.6065
Bilateral (%)	104 (73.76)	30 (69.77)	
Chemotherapy			
No (%)	85 (60.28)	25 (58.14)	0.8018
Yes (%)	56 (39.72)	18 (41.86)	
Endocrine therapy			
No (%)	80 (56.74)	23 (53.49)	0.7071
Yes (%)	61 (43.26)	20 (46.51)	
Radiation			
Yes	17 (12.06)	5 (11.63)	0.9503
No	111 (78.72)	35 (81.4)	
Prior	13 (9.22)	3 (6.98)	
Diagnosis			
Benign	136 (55.51)	33 (45.21)	0.3009
DCIS	25 (10.2)	9 (12.33)	
Invasive cancer	84 (34.29)	31 (42.47)	
Race			
White	123 (87.23)	40 (93.02)	0.7667
African American	10 (7.09)	1 (2.33)	
Hispanic	2 (1.42)	1 (2.33)	
Asian	4 (2.84)	1 (2.33)	
Other	2 (1.42)	0 (0)	

*Note:* Significance was determined at an alpha level of 0.05.

Abbreviations: ASA, American Society of Anesthesiology; BMI, body mass index; DCIS, ductal carcinoma in situ.

**Table 2:** Type of reconstruction.

First step of reconstruction	Average-volume NSM (< 600 g)	Extreme NSM (> 600 g)	*p* value
Expander	58 (41.13)	12 (27.19)	0.2773
Direct to implant	59 (41.84)	23 (53.49)	
DIEP	24 (17.02)	8 (18.6)	

**Table 3:** Total, major, and minor complications following average-volume NSM versus extreme NSM with immediate reconstruction.

	Average-volume NSM (%)	Extreme NSM (%)	*p* value
Complications (all patients)			
No	110/141 (78.01)	25/43 (58.14)	**0.009852**
Yes	31/141 (21.99)	18/43 (41.86)	
Complications (all patients)			
None	110/141 (78.57)	25/43 (58.14)	**0.02841**
Minor	13/141 (9.29)	6/43 (13.95)	
Major	18/141 (12.86)	12/43 (27.91)	
Major versus no complication			
None	110/128 (85.94)	25/37 (67.57)	**0.01072**
Major	18/128 (14.06)	12/37 (32.43)	
Minor versus no complication			
None	110/123 (89.43)	25/37 (80.65)	0.2205
Minor	13/123 (10.57)	6/37 (19.35)	

*Note:* Significance was determined at an alpha level of 0.05.

**Table 4:** Complications following average-volume NSM versus extreme NSM with immediate reconstruction.

Complication	Average-volume NSM number of patients (%)	Extreme NSM number of patients (%)
Seroma	11 (7.80)	7 (16.2)
Hematoma	3 (2.12)	3 (6.98)
Skin necrosis	3 (2.12)	3 (6.98)
Nipple necrosis	0 (0)	2 (4.65)
Cellulitis	11 (7.80)	3 (6.98)
Wound dehiscence	1 (0.79)	2 (4.65)
Wound infection	2 (1.42)	2 (4.65)
Partial flap necrosis	2 (1.42)	1 (2.33)
Total flap necrosis	1 (0.79)	0 (0)
Implant extrusion	2 (1.42)	3 (6.98)

## Data Availability

The data that support the findings of this study are available on request from the corresponding author. The data are not publicly available due to privacy or ethical restrictions.
